# microRNA as a new immune-regulatory agent in breast milk

**DOI:** 10.1186/1758-907X-1-7

**Published:** 2010-03-01

**Authors:** Nobuyoshi Kosaka, Hirohisa Izumi, Kazunori Sekine, Takahiro Ochiya

**Affiliations:** 1 Section for Studies on Metastasis, National Cancer Center Research Institute, 1-1, Tsukiji 5-chome, Chuo-ku, Tokyo 104-0045, Japan; 2 Nutritional Science Laboratory, Morinaga Milk Industry Co., Ltd 1-83, 5-chome, Higashihara, Zama, Kanagawa 228-8583, Japan

## Abstract

**Background:**

Breast milk is a complex liquid that provides nutrition to the infant and facilitates the maturation of the infant's immune system. Recent studies indicated that microRNA (miRNA) exists in human body fluid. Because miRNAs are known to regulate various immune systems, we hypothesized that human breast milk contains miRNAs that may be important for the development of the infant's immune system.

**Findings:**

We profiled miRNA expression in human breast milk and detected high expression levels of immune-related miRNAs in the first 6 months of lactation. Furthermore, these miRNA molecules are stable even in very acidic conditions, indicating that breast milk allows dietary intake of miRNAs by infants.

**Conclusions:**

Our findings provide new insight into how breast milk can modulate the development of the infant's immune system. This study suggests the transfer of genetic material as miRNA from human to human occurs by means other than through sexual reproduction.

## Introduction

The mammary glands of mammals are specialized organs whose function is to produce milk, the primary source of nutrition for newborns. Breastfeeding is recognized as one of the most valuable contributors to infant health [1]. Human breast milk protects infants not only against infections but also against chronic diseases. Furthermore, human breast milk contains certain growth factors that help the infant intestine to develop, become able to absorb milk and prepare for food intake. When maternal breast milk is unavailable, the alternative is infant formula. Compared with infants fed on formula, infants fed on breast milk have a lower incidence of digestive problems and are more likely to be protected against gastrointestinal and respiratory infections. Despite the fact that breastfeeding is known to be the best method for nourishing infants, how exactly breastfeeding works to provide the best nutrition and protect infants against disease is not fully understood.

Many immune-related substances are present in human breast milk, and their effects upon the recipient infants are widely recognized [2,3]. For instance, human breast milk contains large quantities of secretory (s)IgA. These antibodies can bind to pathogens and prevent their attachment to an infant's cells. Furthermore, human breast milk contains measurable levels of leukocytes. In addition to these immunologic components, breast milk contains several nonspecific factors, such as lysozyme, lactoferrin and oligosaccharides, which have antimicrobial effects. Lysozyme inhibits the growth of many bacterial species by disrupting the proteoglycan layer of the bacterial cell wall. Lactoferrin, known as a multifunctional protein in human breast milk, also limits bacterial growth by removing essential iron and by stimulating cytokine production, and enhancing mucosal immunity, natural killer (NK) cell activity and macrophage cytotoxicity. Substantial amounts of oligosaccharides in the mammary gland were found in human breast milk, and these block attachment of microbes to an infant's mucosa, preventing infections. Nucleotides in human breast milk have been shown to enhance the immune function in infants [4]. However, several additional immune regulatory components in milk may explain why breastfeeding can reduce infant mortality.

MicroRNAs (miRNAs) are small regulatory RNA molecules that modulate the activity of specific mRNA targets and play important roles in a wide range of physiologic and pathologic processes [5-7]. Specific miRNAs that play important roles in a wide range of physiologic and pathologic processes in mammals may be involved in the control of immunologic reactions [6,7]. A loss-of-function approach indicated that miRNAs are crucially involved in the *in vivo *control of immune regulation including cellular differentiation and immune response [8-10]. Recently, miRNAs have been found in serum, plasma and other body fluids [11-13]. Serum miRNAs can serve as potential biomarkers for the detection of various cancers and other diseases. Furthermore, it was reported that miRNAs circulate in plasma microvesicles in peripheral blood in healthy people [14]. The majority of the plasma microvesicles from normal individuals are derived from blood cells. However, the physiologic roles of body fluid miRNAs are undetermined. In this report, we show that a considerable number of miRNAs, especially those that function in the immune system, are found in human breast milk. Furthermore, we detected a higher expression of immune-related miRNAs in the first 6 months of lactation. These miRNAs are stable even under harsh conditions. Our findings suggest that human breast milk contains miRNAs capable of transfer to immune cells to support the development of an infant's immune system.

## Results

### Extraction of RNAs and expression analysis

**Figure 1:**
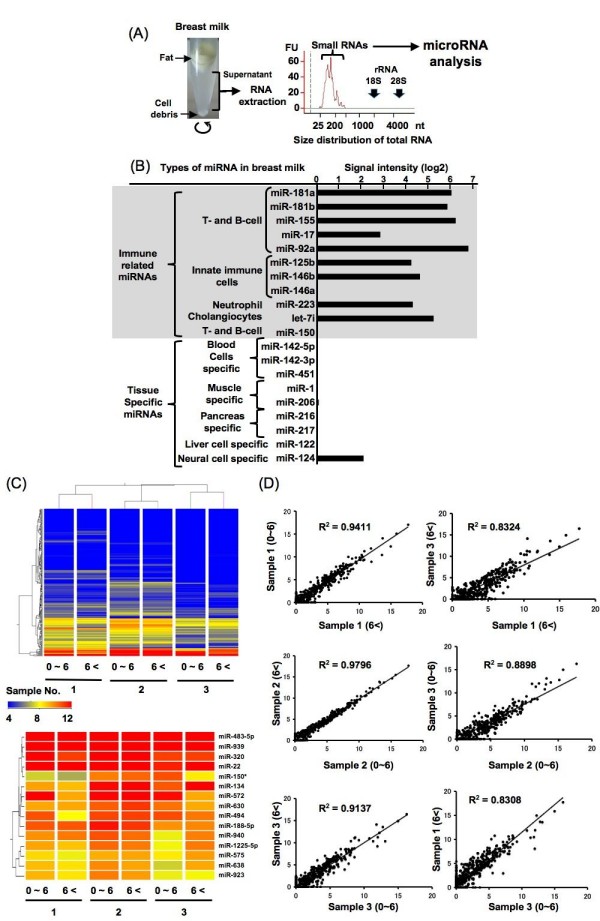
**Expression profile of miRNAs in human breast milk**. **(a) **RNA isolation from breast milk. RNA from breast milk was detected using a Bioanalyzer. The milk contains a substantial amount of small RNA (<300 nt), but very little or no ribosomal RNA (18S and 28S rRNA). **(b--d**) Expression profiling of miRNAs in human breast milk from miRNA microarray. **(b) **miRNAs in breast milk that were detected or hardly detected by microarray analysis. **(c) **Upper panel: a clustering analysis of the expression of breast milk miRNAs before and after the infants received complementary food. Samples 1, 2 and 3 were from different mothers. Breast milk samples were taken during the first 6 months after birth (0--6) and during the subsequent months (6+). Lower panel: Strongly expressed miRNA in breast milk before and after the infants received complementary food. **(d) **Correlation of gene expression signals between samples obtained from the same mother during the first 6 months and during the following 6 months are shown. The relationship between samples from different mothers was not as close.

To verify the existence of miRNAs in human breast milk, we extracted total RNA from human breast milk. miRNAs were detected in each individual at concentrations ranging from 9.7 ng/ml to 228.2 ng/ml. The samples contained a substantial amount of RNA, but no or only very low amounts of ribosomal RNA (18S and 28S) (Figure 1a). Furthermore, large amounts of small RNA (<300 nucleotides) were detected in the milk.

**Figure 2:**
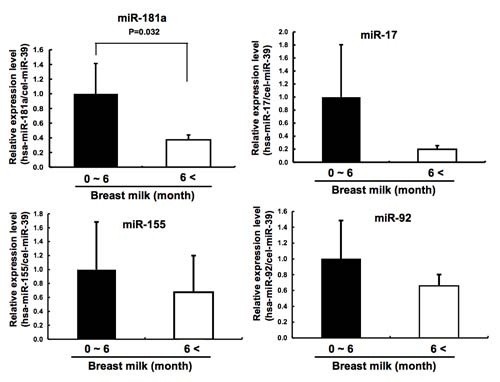
**Comparison by qRT-PCR of immune-related miRNAs in the breast milk obtained before 6 months of age (n = 5) (before the infants received complementary food) and from 6 to 12 months of age (n = 13) (when infants were receiving complementary food)**.

Next we performed a miRNA microarray to show the existence of miRNAs in the milk. Using microarray analysis, 281 of 723 known human miRNAs were detected. Interestingly, several immune-related miRNAs were abundant in the milk (Figure 1b): miR-155, a regulator of T- and B-cell maturation and the innate immune response; miR-181a and miR-181b, regulators of B-cell differentiation and CD4+ T-cell selection; miR-17 and miR-92 cluster: a ubiquitous regulator of B-cell, T-cell and monocyte development, miR-125b, a negative regulator of tumor necrosis factor-α production, activation and sensitivity; miR-146b, a negative regulator of the innate immune response; miR-223, a regulator of neutrophil proliferation and activation; and let-7i, a regulator of Toll-like receptor 4 expression in human cholangiocytes. By contrast, T- and B-cell regulatory miR-150 was not detected, and several tissue-specific miRNAs, such as miR-122 (liver), miR-216, miR-217 (pancreas) and miR-142-5p and miR-142-3p (hematopoietic cells), were barely detectable (Figure 1b). Furthermore, the expression pattern of miRNA within different breast milk samples from the same mother did not differ greatly with time after birth (Figure 1c, d). Between individuals, however, there was more variation. Notably, we detected higher expression of several immune system-related miRNAs from human breast milk in the first 6 months, which is the period before infants receive complementary food (Figure 2).

### Resistance and stability of miRNAs

The existence of RNase in body fluids is already known [13], and thus there should be no intact RNA present. The presence of miRNAs suggests that these miRNA species are resistant to RNase digestion. To prove this possibility, human breast milk was treated with RNase A/T.

**Figure 3:**
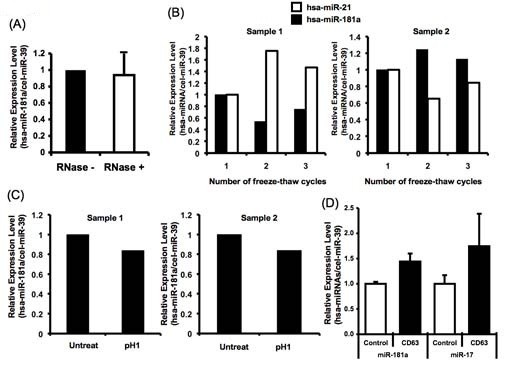
**General characterization of miRNAs in breast milk**. **(a--c) **The stability of breast milk miRNAs. Total RNA was extracted and then analyzed by qRT-PCR. Breast milk was **(a) **incubated with RNase A/T for 3 h at 37°C, **(b) **subjected to three freeze-thaw cycles or **(c) **treated for 3 h in a low pH solution (pH 1) before RNA extraction. Representative data are shown. **(d) **Expression of miR-181a and miR-17 derived from CD63-positive exosomes isolated from human breast milk (0.3 ml) from different mothers (n = 4). Human breast milk was immunoprecipitated with anti-CD63 antibody or isotype control.

Strikingly, treatment with RNase had hardly any effect on human breast milk miRNAs (Figure 3a). By contrast, the profile of total RNA from that milk and exogenously added synthetic miRNAs were degraded by the same treatment (data not shown). These data clearly demonstrate that human breast milk miRNAs are resistant to RNase digestion.

Their stability was further studied after treatment under harsh conditions including freeze-thaw cycles and low pH. The samples were analyzed by quantitative reverse transcription (qRT)-PCR analysis.

Results of qRT-PCR analysis of miRNAs in human breast milk samples subjected to harsh conditions were not significantly different from samples not subjected to these conditions. As shown in Figure 3b, human breast milk miRNAs were resistant to several freeze-thaw cycles. Moreover, human breast milk miRNAs were stable when the milk was treated for 1 h in an acidic (pH 1) solution (Figure 3c).

### Existence of microvesicles

Observation of the stability of human breast milk miRNAs *in vitro *suggests the possibility that they are contained within microvesicles or exosomes [14]. To verify this, we isolated the CD63-positive exosome fraction and investigated miRNA expression.

As shown in Figure 3d, miR-181a and miR-17, which we detected by microarray analysis, were also detected in the CD63-positive exosome fraction. However, it is still possible that other miRNA protection mechanisms, such as an apoptotic body, may protect miRNA from harsh conditions.

### Presence of miRNAs in other body fluids

It was previously reported that miRNAs are also detected in serum samples [11-13]. To investigate differences in miRNA expression in different body fluids, we analyzed expression of several immune-regulated miRNAs in human serum and human breast milk.

**Figure 4:**
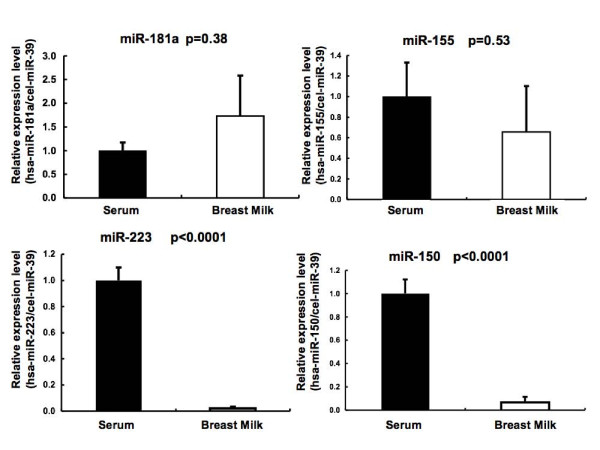
**Immune-related miRNA levels in human breast milk (n = 5) and serum (n = 6) of healthy subjects measured by qRT-PCR**.

Interestingly, the granulocyte-regulated miRNA, miR-223, is the most strongly expressed miRNA in normal human plasma, but its expression in breast milk is low compared with that in serum (Figure 4) [14]. On the other hand, breast milk is rich in miR-181 and miR-155, and these were detected at similar levels in serum. Furthermore, miRNA microarray analysis revealed that expression of miR-146a, which is high in plasma, was not found in breast milk, whereas miR-146b, which is not abundant in plasma was abundantly expressed in breast milk (Figure 1b).

## Discussion

There are several reports in the literature showing the presence of miRNAs in body fluids including blood plasma/serum, saliva and urine [11-13]. These studies showed the usefulness of miRNAs as a biomarker for disease, but did not investigate any biologic function of the miRNAs in body fluid. Furthermore, because the function of breast milk is to provide nutrition to infants from mothers, miRNA could also have functions in an infant's body.

In our study, miRNAs in breast milk were stable even under very acidic conditions (pH 1). This suggests that these molecules can tolerate an infant's gastrointestinal environment and be absorbed in to the intestine, thus influencing the immune system, as the intestine is one of their major immune organs (Figure 3c). The storage and freeze-thawing of breast milk did not denature the miRNAs, a dietetically important finding for low-birthweight babies and other hospitalized infants who are usually given freezer-stored breast milk (Figure 3b). The resistance of the miRNAs to the RNase treatment indicated that the miRNAs in human breast milk might be packaged inside complexes such as exosomes or microvesicles (Figure 3a) [14,15]. Furthermore, as shown in Figure 3d, we detected miR-181a and miR-17 in a CD63-positive fraction from human breast milk. A previous report suggested that breast milk-derived exosomes can increase the number of Foxp3+ CD4+ CD25+ regulatory T cells in infants [16]. This is consistent with our results, showing that human breast milk is rich in T-cell-regulating miRNAs [17,18]. Furthermore, human breast milk miRNAs may induce B-cell differentiation, because the milk is rich in miR-181 and miR-155, both known to induce B-cell differentiation [19,20], but it is not rich in miR-150, which suppresses B-cell differentiation [21,22]. Our miRNA microarray detected many different types of miRNAs in human breast milk (Figure 1c); however, the functions of many of these are still unknown. Because immune-related miRNAs are well studied and their functions are clarified in the present report, we focused on their presence in human breast milk in which we believe miRNAs should have many more functions, especially in immunologic conditions such as allergy, including atopy and asthma [23,24].

**Figure 5:**
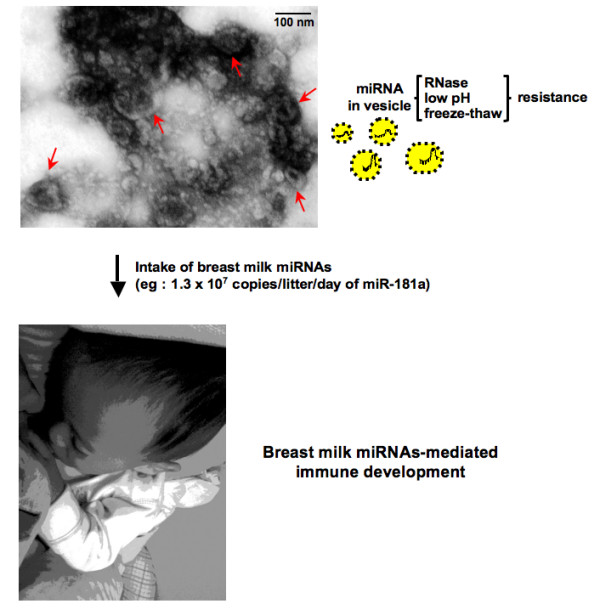
**Exosome-like vesicles from human breast milk display the typical size (30--300 nm) and ultrastructure of electron-dense and electron-lucent microvesicles (arrows)**. miRNAs in the vesicles within breast milk are received by infants and facilitate many aspects of the infant's development. Scale bar, 100 nm.

The transfer of miRNA among cells means that miRNA is not only a regulatory molecule within the cell, but also, like cytokines, is a regulatory molecule for cell-cell communication. Our study clearly suggests that miRNA is a transferable genetic material from mother to infant. It is estimated that approximately 1.3 × 10^7 ^copies/liter/day of miR-181a are received by a breastfed infant. Further studies are needed to examine the potential clinical use of immune-related miRNAs in breast milk and the mechanisms by which these miRNAs act as a tool for molecular communication between mother and infant (Figure 5). As shown in Figure 1c, although there was more variation between individuals, the expression pattern of miRNA in human breast milk samples from the same mother did not differ much with time after birth. These observations suggest that human breast milk reflects a mother's constitution and her living environment such as food intake and climate. The present study provides insight into how breast milk may protect infants from various infections; proposing that the miRNAs there act as immune-regulatory agents.

## Materials and methods

### Ethics statement

All the women gave their signed informed consent to participate. The study was approved by Dr. Tadao Ishii, Manager of Morinaga Co., Ltd and the company's ethics committee (the Ethical Committee of Functional Food Creation).

### Sample collection

Human breast milk samples were collected from eight women enrolled in a breastfeeding study at Morinaga Milk Industry Co., Ltd. Human breast milk samples were collected when the infant were aged between 4 days and 11 months (see Table 1). The milk was collected and then the collected samples totaling 50-100 ml were put into in storage bags. All samples were stored at - 80°C until analyzed.

**Table 1:** Time of sample collection

Volunteer	Sample collection (months after birth)
1	6, 8, 8
2	4, 6, 7
3	6, 7, 8, 8
4	7, 7, 8, 9
5	9, 10
6	4 samples within 1 month
7	No information
8	2, 4, 10, 12

### Sample preparation

We chose the samples given <6 months after birth and samples given between 6 and 12 months after birth. Cells and large debris within the breast milk were removed by centrifugation at 2,000 × *g *for 10 min twice; the supernatant was then centrifuged at 12,000 × *g *for 30 min to remove cellular debris. The clear supernatants were used for the analysis.

### Total RNA extraction

Total RNA from breast milk was extracted using a mirVana miRNA isolation kit (Ambion, Austin, TX, USA). Breast milk was thawed on ice, diluted with two volumes of *mir*Vana Lysis/Binding Solution, mixed thoroughly by vortex for 30 s and incubated for 5 min. Then 1/10 volumes of miRNA homogenate additive was added, mixed thoroughly by vortex for 30 s and incubated on ice for 10 min. An equal volume of acid/phenol/chloroform (Ambion) was then added to each aliquot. The resulting solutions were mixed by vortex for 1 min and spun for 10 min at 10,000 × *g*. The resulting aqueous volume was mixed thoroughly with 1.25 volumes of 100% molecular-grade ethanol and passed through a *mir*Vana column in sequential 700 μl aliquots. The column was washed according to the manufacturer's protocol, and RNA was eluted in nuclease-free water at 95°C. RNA extraction from the serum was performed using the same method.

### Microarray analysis

To detect the expression of miRNAs in human breast milk, 70 ng of total RNA was labeled and hybridized using a Human microRNA Microarray Kit (Agilent Technologies) according to the manufacturer's protocol (Protocol for Use with Agilent Microrna Microarrays Version 1.5). Hybridization signals were detected by a DNA microarray scanner (Agilent Technologies), and the scanned images were analyzed using Agilent Feature Extraction software.

#### qRT-PCR

qRT-PCR of miRNA expression was performed using the TaqMan MicroRNA Assay (Applied Biosystems, Foster City, CA, USA), according to the manufacturer's protocol. To normalize the sample-to-sample variation in the RNA isolation step, synthetic *Caenorhabtidis elegans *miRNA cel-miR-39 (synthetic RNA oligonucleotides synthesized by Qiagen, Valencia, CA, USA) was added as a mixture of 25 fmol of each oligonucleotide in a total volume of 1 ml to each denatured sample (that is, after combining the breast milk and serum samples with Lysis Solution (mirVana miRNA isolation kit; Ambion). All experiments were repeated three times.

### RNase and freeze-thawing treatment

To confirm that the miRNA is resistant to RNase digestion, human breast milk was treated with 10 U/ml RNase A and 400 U/ml RNase T1 (Ambion) for 60 min at 37°C. After these treatments, the RNA was extracted from the milk as described above.

To investigate the stability of miRNAs in human breast milk, the milk was subjected to three freeze-thaw cycles of at -20°C or treated for 3 h in a low pH solution (pH 1). miRNA levels were assessed by TaqMan qRT-PCR.

### Isolation of CD63 positive exosome

Exosomes in human breast milk were specifically isolated by magnetic beads, using anti-CD63 antibody (BD, Erembodgem, Belgium). Human breast milk (0.3 ml) was incubated with anti-CD63 antibody (BD) or mouse IgG1 (Sigma-Aldrich, St Louis, MO, USA) coupled to magnetic microbeads (50 μl). These were mixed and incubated for 16 h at room temperature. The magnetic immune complexes were washed four times with 500 μl of PBS, then the RNA was extracted as described above.

### Transmission electron microscopy

Clear supernatants from human breast milk were centrifuged at 100,000 × *g *for two hours, then washed in phosphate-buffered saline (PBS) and pelleted by ultracentrifugation (100,000 × *g*). The pellet was diluted in PBS. Resuspended exosomes were fixed in 1% glutaraldehyde in PBS (pH 7.4). The samples were stained for 10 min with 1% uranyl acetate. Excess fluid was removed with a piece of Whatman filter paper. All transmission electron micrographs were obtained using JEM1220 electron microscopy at 120 kv.

## Competing interests

The Morinaga Milk Industry Co., Ltd is a commercial for-profit company. HI and KS are employed/funded by salaries from Moringa Milk Industry Co., Ltd. The Morinaga Milk Industry Co., Ltd has filed patent applications on aspects of this work. NK, KS, HI and TO are named as inventors with the Morinaga Milk Industry Co., Ltd on patent application number 2009-165991/July14, 2009.

## Authors' contributions

NK designed and performed the experiments and wrote the manuscript. HI and KS prepared the samples and helped to draft the manuscript. TO designed experiments and wrote the manuscript. All authors read and approved the final manuscript.
